# Meat Feeding Restricts Rapid Cold Hardening Response and Increases Thermal Activity Thresholds of Adult Blow Flies, *Calliphora vicina* (Diptera: Calliphoridae)

**DOI:** 10.1371/journal.pone.0131301

**Published:** 2015-07-21

**Authors:** Paul C. Coleman, Jeffrey S. Bale, Scott A. L. Hayward

**Affiliations:** School of Biosciences, University of Birmingham, Birmingham, United Kingdom; New Mexico State University, UNITED STATES

## Abstract

Virtually all temperate insects survive the winter by entering a physiological state of reduced metabolic activity termed diapause. However, there is increasing evidence that climate change is disrupting the diapause response resulting in non-diapause life stages encountering periods of winter cold. This is a significant problem for adult life stages in particular, as they must remain mobile, periodically feed, and potentially initiate reproductive development at a time when resources should be diverted to enhance stress tolerance. Here we present the first evidence of protein/meat feeding restricting rapid cold hardening (RCH) ability and increasing low temperature activity thresholds. No RCH response was noted in adult female blow flies (*Calliphora vicina* Robineau-Desvoidy) fed a sugar, water and liver (SWL) diet, while a strong RCH response was seen in females fed a diet of sugar and water (SW) only. The RCH response in SW flies was induced at temperatures as high as 10°C, but was strongest following 3h at 0°C. The CT_min_ (loss of coordinated movement) and chill coma (final appendage twitch) temperature of SWL females (-0.3 ± 0.5°C and -4.9 ± 0.5°C, respectively) was significantly higher than for SW females (-3.2 ± 0.8°C and -8.5 ± 0.6°C). We confirmed this was not directly the result of altered extracellular K^+^, as activity thresholds of alanine-fed adults were not significantly different from SW flies. Instead we suggest the loss of cold tolerance is more likely the result of diverting resource allocation to egg development. Between 2009 and 2013 winter air temperatures in Birmingham, UK, fell below the CTmin of SW and SWL flies on 63 and 195 days, respectively, suggesting differential exposure to chill injury depending on whether adults had access to meat or not. We conclude that disruption of diapause could significantly impact on winter survival through loss of synchrony in the timing of active feeding and reproductive development with favourable temperature conditions.

## Introduction

To cope with winter most temperate insects enter a period of dormancy termed diapause [1]. Diapause is typically characterised by a sequestration of nutrient reserves, suppression of activity and metabolism, an arrest or slowing of development and increased tolerance to environmental stress [2]. There is increasing evidence, however, that climate change is disrupting the diapause program [3]. For example, in the blow fly *Calliphora vicina*, diapause is induced when maternal adults detect a specific photoperiod in late-autumn, termed the critical day length (CDL) [4]. Diapause is then initiated by third instar larvae of the subsequent generation as long as temperatures do not exceed 20°C for adults, or 15°C for larvae [5,6]. A disrupting effect of warmer autumns on diapause incidence has been noted in other species [3,7], and given the widespread maternal induction of diapause [8], this phenomena is likely to impact a substantial number of temperate insects. For some species, warmer conditions bring the benefit of longer growing seasons and the opportunity to expand population size and spatial distribution [9,10]. For others, however, comes the increased risk of non-diapause (active and feeding) life stages being exposed to winter cold [7,11].

For species such as *C*. *vicina*, if diapause is averted in late autumn, the adult stage cannot persist for the entire winter. This poses a number of conflicting problems, including the need to feed and remain mobile to forage, whilst also enhancing cold tolerance. Feeding is not conducive to winter survival, however, for a number of reasons. Most insects studied to date cannot survive freezing [12], including *C*. *vicina* [13], and many evacuate their gut prior to overwintering or as a direct response to cold [14,15]. This is because their freezing temperature, the supercooling point (SCP), is strongly influenced by the presence of ice nucleating agents (INAs) such as food particles [16]. Indeed, there is strong evidence that the type of food consumed can directly influence SCPs [6]. Even where freezing is not a risk, significant mortality can occur due to cold injuries well above the SCP [17]. Insects can increase their cold tolerance through gradual acclimation or rapid cold hardening (RCH) [3]. The latter representing the ability to enhance survival of extreme cold through brief (minutes to a few hours) exposure to a less severe temperature [17,18]. A RCH response has been identified in many species, including some that naturally lack the ability to enter diapause [11,19] and is a reversible response that can occur during any developmental stage or at any time of the year [20]. Consequently, it is highly relevant to life stages active during winter, and potentially exposed to significant diurnal temperature fluctuations. The physiological effects of feeding are further complicated by the fact that adult feeding is often sufficient to switch resource allocation towards reproductive development [4,21], thus diverting it away from stress response processes. For example, in *C*. *vicina* and *C*. *erythrocephala*, meat/protein feeding initiates egg development [4], while for the black blow fly, *Phormia regina*, it instigates reproductive gland development [21]. How adult feeding might influence RCH, however, has never been studied.

As well as influencing cold survival, feeding can also alter thermal activity thresholds. For all insects there is a defined thermal window of activity, and at temperatures outside of this range, individuals become vulnerable to starvation and predation as well as chill-induced mortality [22,23]. As insects are cooled, activity becomes impeded and walking speed decreases until the ability to maintain coordinated movement is lost, termed the critical thermal minima (CT_min_). Below this temperature, the individual eventually reaches a point of chill coma—a reversible physiological state signified by a final appendage twitch [22]. The CT_min_ in particular is recognised as an ecologically relevant measure of cold tolerance and a useful trait in predicting ectotherm geographic distributions [24,25]. The dominant hypothesis regarding the causes of chill coma is linked to loss of ion homoestasis during cooling, particularly increased extracellular K^+^, resulting in depolarized muscle membrane potential (Vm) and loss of muscle excitability [26]. Dietary K^+^ has also previously been linked to impaired movement [27], and given the high K^+^ content of meat, the CT_min_ and/or chill coma temperature of winter active *C*. *vicina* adults could be altered by feeding. Interestingly, dietary K+ did not alter chill coma temperatures in the locust, *Locusta migratoria*, though it did influence chill coma recovery (CCR) [28]. The complex diet in this latter study, however, would have altered much more than just extracellular K+. One way to disentangle this complexity is to simply supplement the diet of insects with alanine, which causes K^+^ efflux in animal cells [29], and then examine the effects of this ion imbalance on cold tolerance.

Against this background, the current study investigated the effect of meat feeding on the: (1) RCH response (2) ability to acclimate (3) thermal activity thresholds, and (4) supercooling capacity of *C*. *vicina* adults. To specifically address whether K^+^ ion imbalance alone contributed to differences in cold tolerance, we also supplemented the diet of flies with alanine. Finally, *in-situ* temperature data was used to calculate the frequency (number of days) that temperatures were below the CT_min_ threshold over the winter period in Birmingham, UK (52.4°N, 1.9°W), to determine the risk of diapause disruption on population persistence.

## Materials and Methods

### Experimental flies

*Calliphora vicina* used in this study were originally sourced from the University of Birmingham campus, Birmingham, UK (52.4°N, 1.9°W) in 2009 using olfactory-traps [30]. Lab cultures were regularly replenished with wild caught individuals to prevent inbreeding [7].

Female cultures were established on the day of mass eclosion under photoperiodic and temperature conditions typical of late-autumn/ early-winter (LD 12:12 h at 20°C). While we recognise some geographic strains of *C*. *vicina* possess the ability to enter a reproductive diapause [31,32], we have identified that Birmingham strain females continue to oviposit at LD12:12 and 10°C. Furthermore, winter active adults in the field fed on meat when temperatures were below 5°C [33]. Thus, we can be certain that no females used in this study were in reproductive diapause.

Females were either fed sugar, water and (pigs) liver (SWL) or just sugar and water (SW). Sugar and water was provided a*d libitum* to ensure that no females were starved or dehydrated. The SWL females received liver on d 4 and 6 post eclosion. The mineral content of swine liver is summarised in Table 1.

**Table 1:** The standard mineral content (mg) of swine liver given per 100g.

Mineral	Content per 100g (mg)
Calcium	26.0
Iron	6.4
Magnesium	12.0
Phosphorus	230.0
Sodium	860.0
Potassium	170.0
Zinc	2.3
Copper	0.24

### The discriminating temperature

The discriminating temperature (DTemp) in RCH experiments is the exposure time and temperature resulting in approximately 80% mortality [34]. This was determined following the plunge method [35]. Groups of ten d 6 females were placed in 50 ml test tubes and plunged into an alcohol bath (Grant LTD D6C, Grant Instruments, UK) set at temperatures ranging from -4°C to -9°C (1°C increments) for 2 h. Survival was measured as the ability to regain movement following 2h at 20°C (6 replicates of *n* = 10 for each treatment group), noting that preliminary experiments found only negligible differences in mortality after 2h and 24h recovery.

### RCH and acclimation

RCH ability was assessed in d 6 SWL and SW females following 3 h at 0°C, 5°C or 10°C, before exposure to DTemp (6 replicates of *n* = 10 for each treatment group).

Survival at the DTemp was also assessed in d 6 SWL and SW females following acclimation to an ecologically relevant thermal cycling regime, ranging from 10°C to 20°C and back over a 24 h period (approximately 1.25°C h ^-1^). [33]. Females were established under these conditions at 10°C (00.00h) on d 1 and removed during the warming phase at 10°C, 15°C and 20°C on d 6 before direct transfer to DTemp (6 replicates of *n* = 10 for each treatment group).

### Supercooling capacity

SCPs were recorded for d 6 SWL and SW flies by attaching individual females to type K exposed wire thermocouples using a small amount of OecoTak A5 (Oecos Ltd, Kimpton, Hertfordshire, UK). Individuals were then placed into 1 ml Eppendorf tubes (Sigma-aldrich, Gillingham, Dorset, UK), which were placed in boiling tubes submerged in a programmable alcohol bath (two tubes per boiling tube) [36]. The temperature was reduced at 0.5°C min^-1^ from 20°C (the culturing temperature) to -30°C, and the SCP was detected by an exothermic output upon freezing. *n *= a minimum of 8 × 3 replicates per treatment.

### CT_min_ and chill coma

The CT_min_ and chill coma of d 6 females were assessed by adapting the methods of Hazell *et al*. [37] using a cooling rate of 0.2°C min^-1^ from 20°C to -15°C. Preliminary experiments confirmed 100% of flies entered chill coma prior to this temperature. Females were placed in a central cooling arena (40 mm in diameter × 7.5 mm in depth) that allowed passage of cooled fluids from an attached alcohol bath (Haake Phoenix 11 P2, Thermo Electron Corp., Karlsruhe, Germany). The arena opening was then covered with a microscope slide (76 × 26 mm). A type K thermocouple connected to a thermometer (Tecpel Advanced Digital Thermometer DTM-315, Heatmiser, UK) was inserted into the arena wall, which recorded the temperature throughout the experiment. Activity was recorded using a digital camera (Infinity 1–1, Lumenera Scientific, Ottawa, Canada) and macro-lens (Computer MLH-10X, CBC Corp., USA), and video recording software (Studio Capture DT, Studio 86 Designs, Lutterworth, UK). Thermal thresholds were determined upon subsequent video playback, with the CT_min_ identified as the temperature at which coordinated movement was lost, and chill coma by a final appendage (typically leg) twitch. This protocol was followed for both SWL and SW females with *n* = a minimum of 13 flies per treatment.

### Alanine supplementation

Newly eclosed females were maintained as outlined for SW females, with the exception of being provided with 112 mM of alanine in the freely accessible water (1 g:100 ml water). Females were then used for experiments on d 6 post-eclosion. Negligible mortality was noted at this dose. The SCP, CT_min_ and chill coma temperatures were determined as described previously.

### The frequency of winter field temperatures below the CT_min_

How frequently Birmingham winter temperatures fell below the CT_min_ of both SW and SWL female *C*. *vicina* was calculated using climate data over the late-autumn/winter period (1^st^ October to the 31^st^ April) from 2009 to 2013. Temperature data were recorded at 1 h intervals using Tinytag Transit Dataloggers (Gemini Data Loggers Ltd, West Sussex U.K.) at a height of 20 cm—the same location as fly traps used to establish lab cultures. The number of days for each month that temperatures fell below the CT_min_ (-3.2°C and -0.3°C for SW and SWL, respectively) were used to produce the frequency data.

### Statistical analysis

Lower thermal activity thresholds had to be calibrated against direct temperature recordings from immobile females held within the cooling arena. Calibrated data were then subjected to a Kolmogorov-Smirnov test to identify the distribution that best described results. All RCH, chill coma and CT_min_ data were normally distributed and so analysed using separate General Linear Models (GLMs) with adult feeding treatment (SWL, SW and alanine fed) and survival as factors, and the significant difference between groups identified using the Bonferroni post-hoc test with an alpha threshold of 0.05. SCP were analysed using the Kruskal-Wallis test (as the requirements for parametric testing were not met). All analyses were performed in SPSS (v. 20.0, IBM, New York, USA) and means are given ± S. E. M.

## Results

### Determination of the DTemp

**Fig 1:**
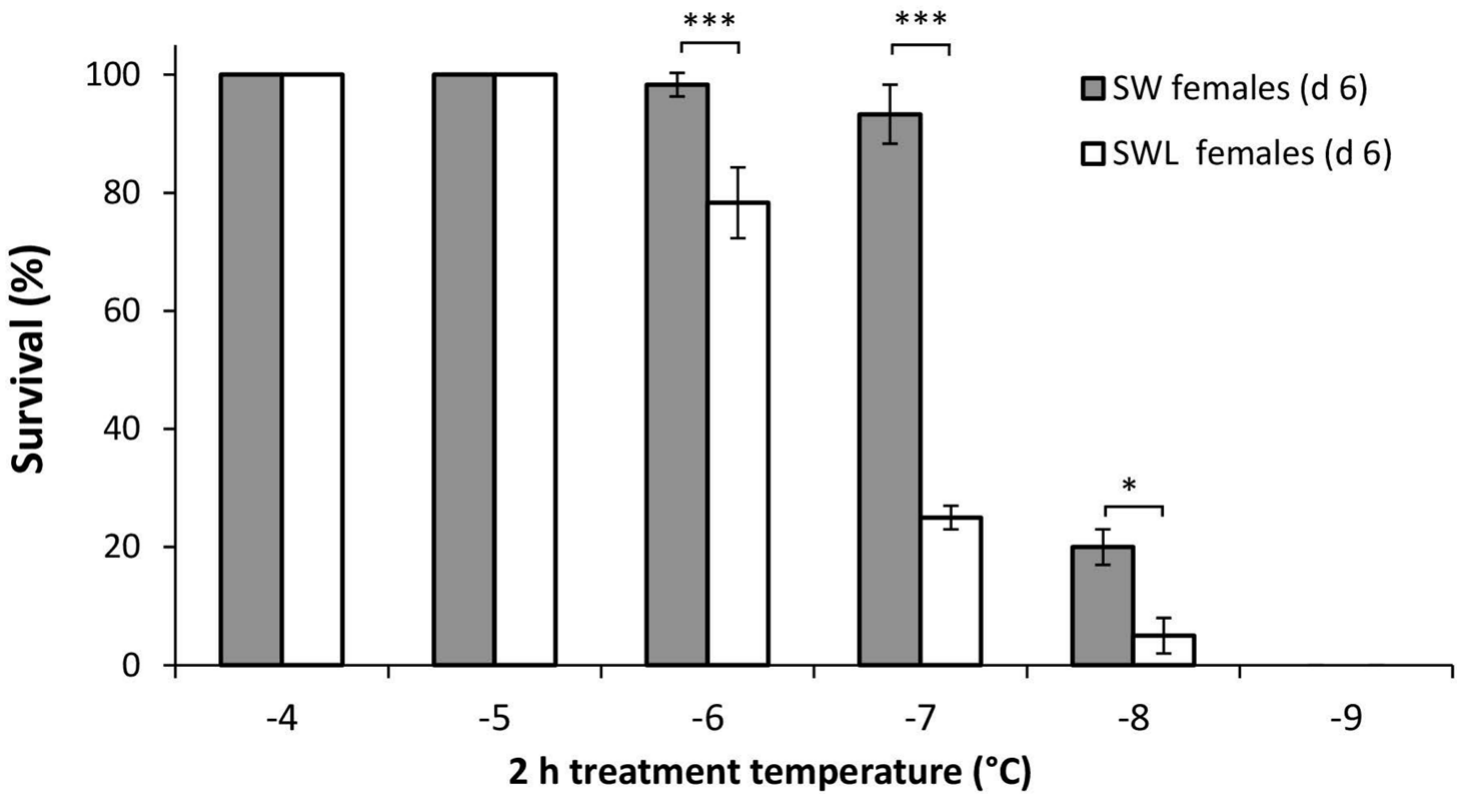
Mean survival (±S. E. M.) of *Calliphora vicina* females either developed on sugar, water and liver (SWL), or sugar and water only (SW), following 2 h exposure to progressively lower sub-zero temperatures (-4° to -9°C). Survival was assessed as spontaneous movement after 2h recovery at 20°C. * denotes significant differences at *p*<0.05, ** at *p*<0.01 and *** at *p*<0.001 (Bonferroni post-hoc). 6 replicates of *n* = 10 for each data point.

There was a significant difference in the cold tolerance of SWL and SW females (F_11,60_ = 297.4, *p*<0.001), with post-hoc analysis revealing SW females were significantly more tolerant of 2 h at -6°C (*p*<0.001), -7°C (*p*<0.001) and -8°C (*p*>0.05) (Fig 1). The DTemp was determined as 2 h at -8°C for SW females (20 ± 2.6% survival) and 2 h at -7°C for SWL females (22 ± 3.1% survival).

### RCH and acclimation

**Fig 2:**
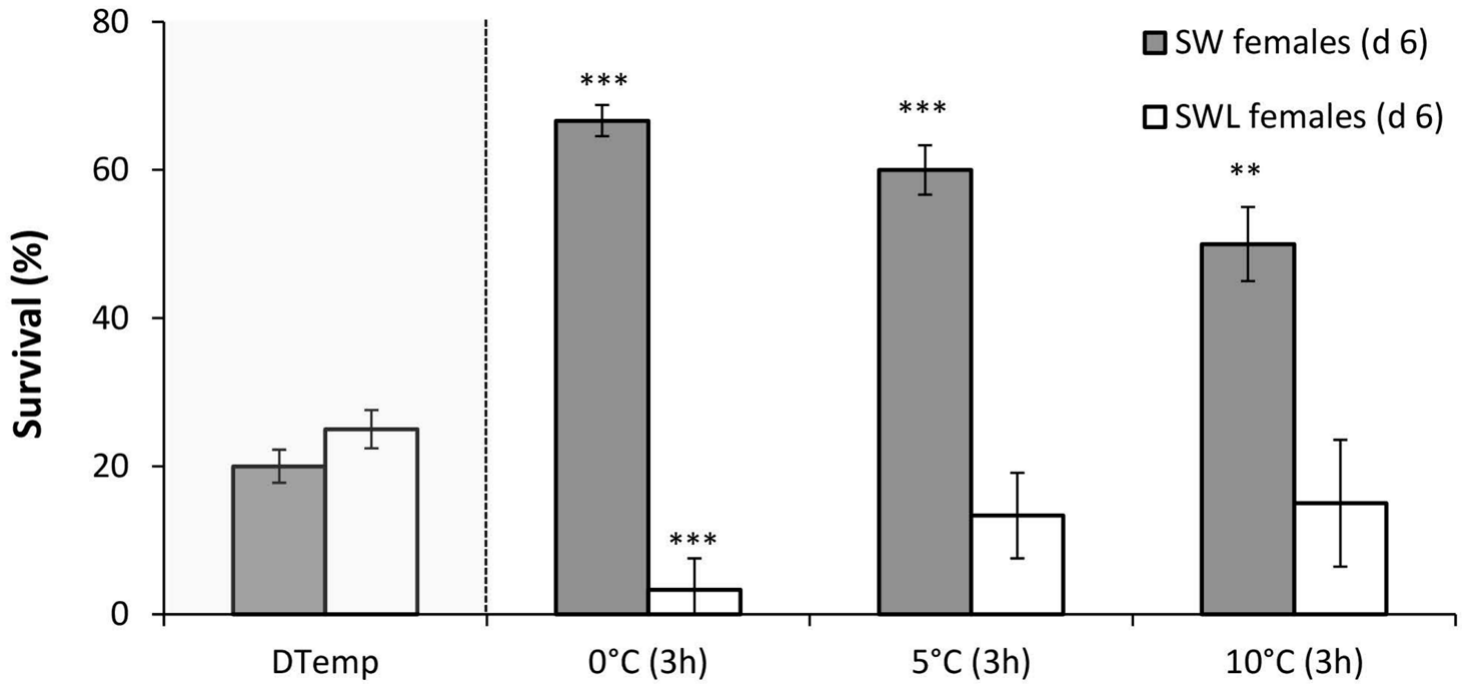
Mean survival (±S. E. M.), as a measure of Rapid Cold Hardening (RCH) ability, of d 6 *Calliphora vicina* females either developed on sugar, water and liver (SWL) or sugar and water (SW) only. RCH was induced using three different constant temperature pre-treatments (3 h at either 0, 5 or 10°C). Survival following RCH was compared with that following direct transfer to respective discriminating temperature (DTemp—in shaded grey area for comparison). * denotes significant differences at *p*<0.05, ** at *p*<0.01 and *** at *p*<0.001 (Bonferroni post-hoc), 6 replicates of *n* = 10 for each data point.

The SW females exhibited a clear RCH response (F_3,23_ = 13.0, *p*<0.001), with significantly enhanced survival at the DTemp following pre-treatments of 3h at 0°C (66.7 ± 4.2%; *p*<0.001), 5°C (60.0 ± 5.8%; *p*<0.001) and 10°C (50.0 ± 8.6%; *p*<0.01) (Fig 2).

The SWL females did not exhibit an increase in total survival at the DTemp following any RCH pre-treatment, however there was a significant decrease in total survival following a pre-treatment of 3h at 0°C (3.3 ± 2.1%; F_3,23_ = 6.9, *p*<0.001).

**Fig 3:**
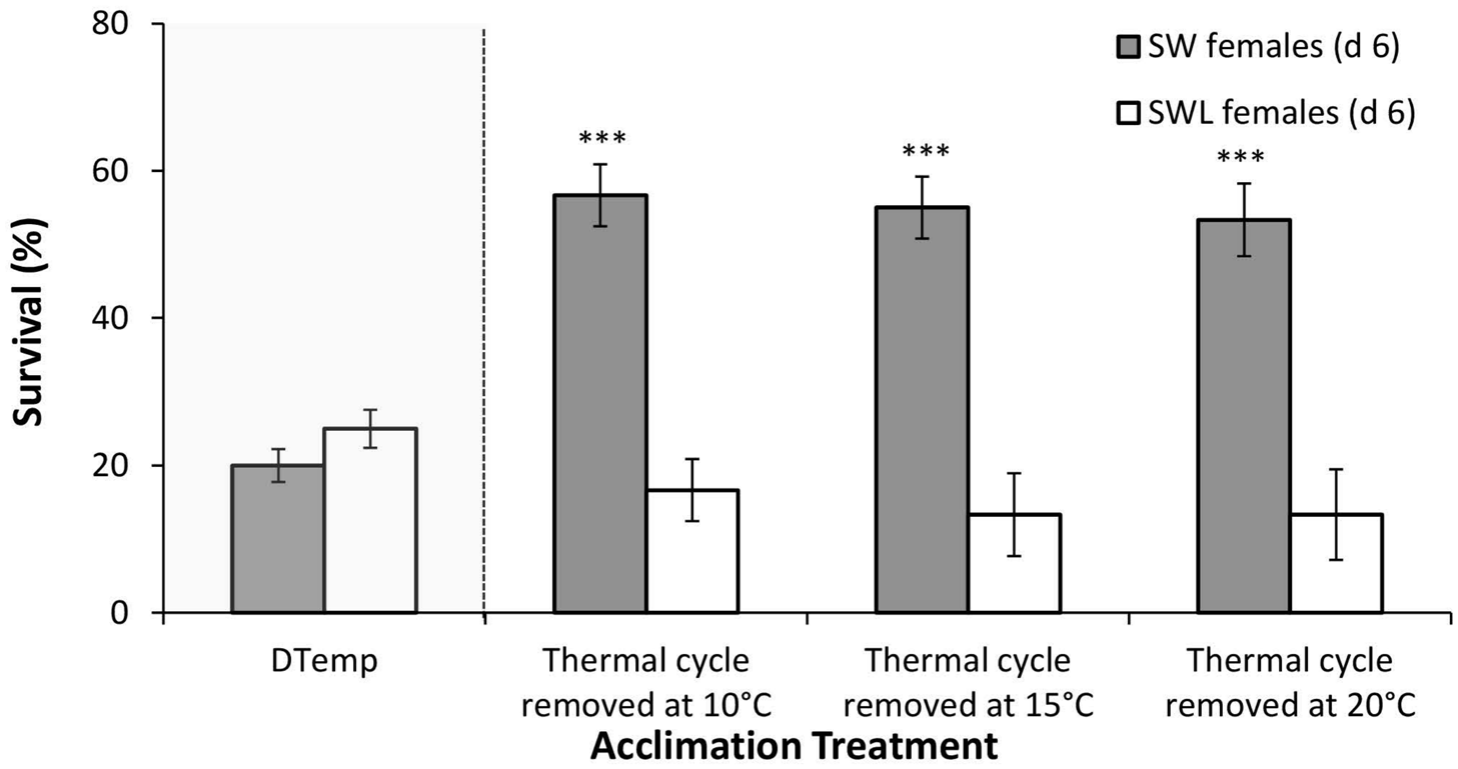
Mean survival (±S. E. M.), as a measure of Rapid Cold Hardening (RCH) ability, of d 6 *Calliphora vicina* females either developed on sugar, water and liver (SWL), or sugar and water (SW) only. RCH was assessed as survival at their respective discriminating temperatures (DTemp—in shaded grey area for comparison) following a 6 d thermal cycle (between 10 and 20°C) at which adults were removed at either 10°C, 15°C or 20°C. * denotes significant differences from DTemp at *p*<0.05, ** at *p*<0.01 and *** at *p*<0.001 (Bonferroni post-hoc). 6 replicates of *n* = 10 for each data point.

Survival at the DTemp increased significantly in SW females following acclimation to the 6 d thermal cycle (F_3,23_ = 13.1, *p*<0.001), with a significant increase in total survival for females removed at 10°C (56.6 ± 4.2%; *p*<0.001), 15°C (55.0 ± 5.6%; *p*<0.001) and 20°C (53.3 ± 6.1%; *p*<0.001) (Fig 3). Survival did not differ significantly between samples removed at the different temperatures during the warming phase.

Increased survival at the DTemp was not detected for SWL females following the 6 d thermal cycle (F_3,23_ = 1.9, *p*<0.168). Survival was consistently higher for SW females compared to SWL females following all treatments.

### Supercooling capacity

The SCPs were not significantly different between SWL (-10.3 ± 0.5°C) SW (-11.0 ± 0.6°C) or alanine fed (-10.0 ± 1.0°C) d 6 females (*n* = 79: Kruskal Wallis test: Chi^2^ = 0.919, *p* = 0.632).

### CT_min_ and chill coma

**Fig 4:**
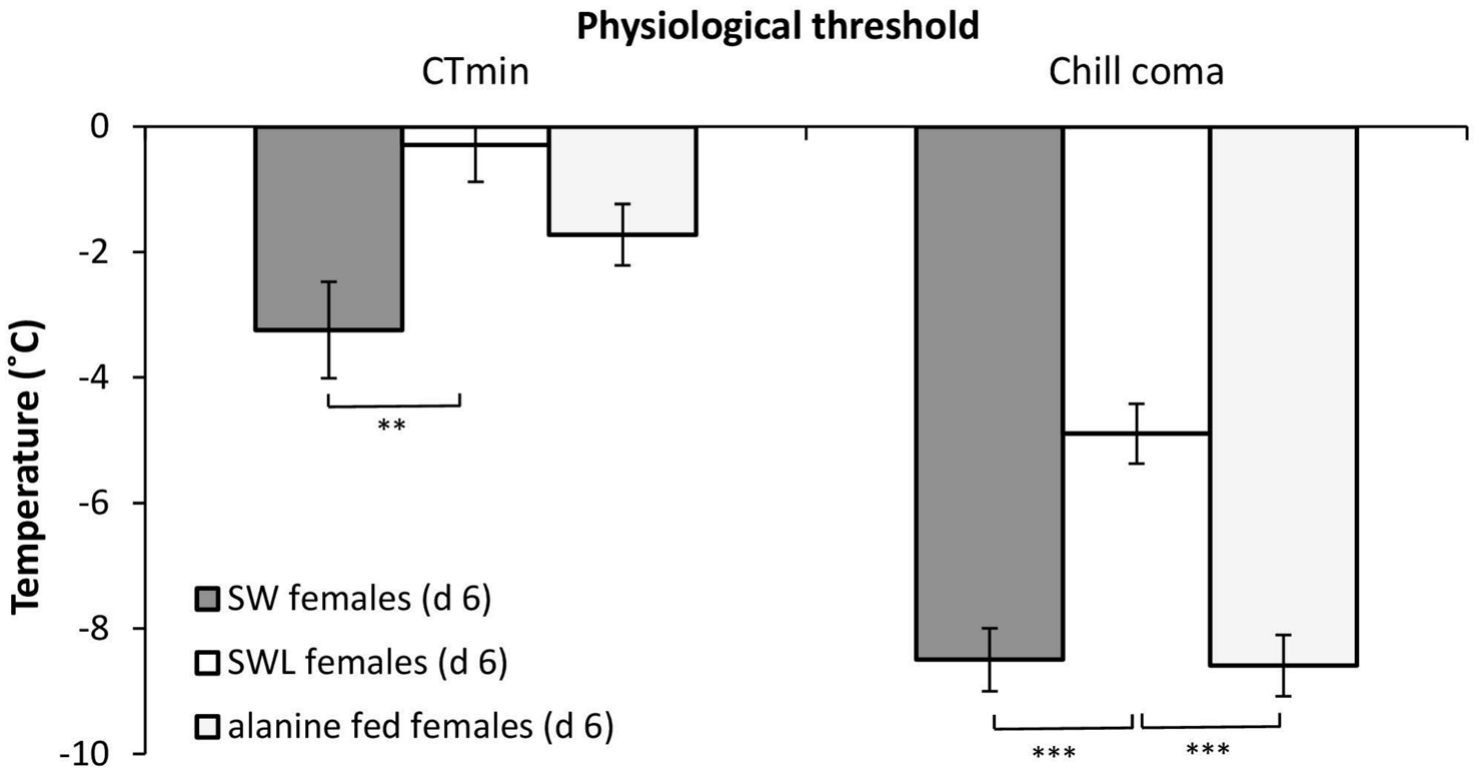
Mean chill coma and CT_min_ temperature (±S. E. M.) of d 6 *Calliphora vicina* females either developed on sugar, water and liver (SWL), sugar and water (SW) only, or sugar and a 112 mM alanine-water solution (1 g:100 ml water). * denotes significant differences at *p*<0.05, ** at *p*<0.01 and *** at *p*<0.001 (Bonferroni post-hoc). *n* = 13 to 17 replicates per data point.

The temperature that females reached CT_min_ was significantly different between groups (F_2,40_ = 6.4, *p*<0.005), with post-hoc analysis identifying a significantly lower CT_min_ for SW (-3.2 ± 0.8°C) than for SWL females (-0.3 ± 0.5°C; *p*<0.005) (Fig 4). The CT_min_ for alanine fed females (-1.7 ± 0.3°C) was intermediate, and not significantly different to either SW (*p* = 0.28) or SWL (*p* = 0.29) females.

The temperature that females reached chill coma was also significantly different between groups (F_2,41_ = 18.9, *p*<0.001). The temperature that SW females reached chill coma (-8.5 ± 0.6°C) was significantly lower than for both SWL (-4.9 ± 0.5°C; *p*<0.001) and alanine fed (-8.6 ± 0.3; *p*<0.001) females (Fig 4). No difference was observed between SW and alanine fed females (*p* = 1.0).

### The frequency of temperatures below the CT_min_

**Fig 5:**
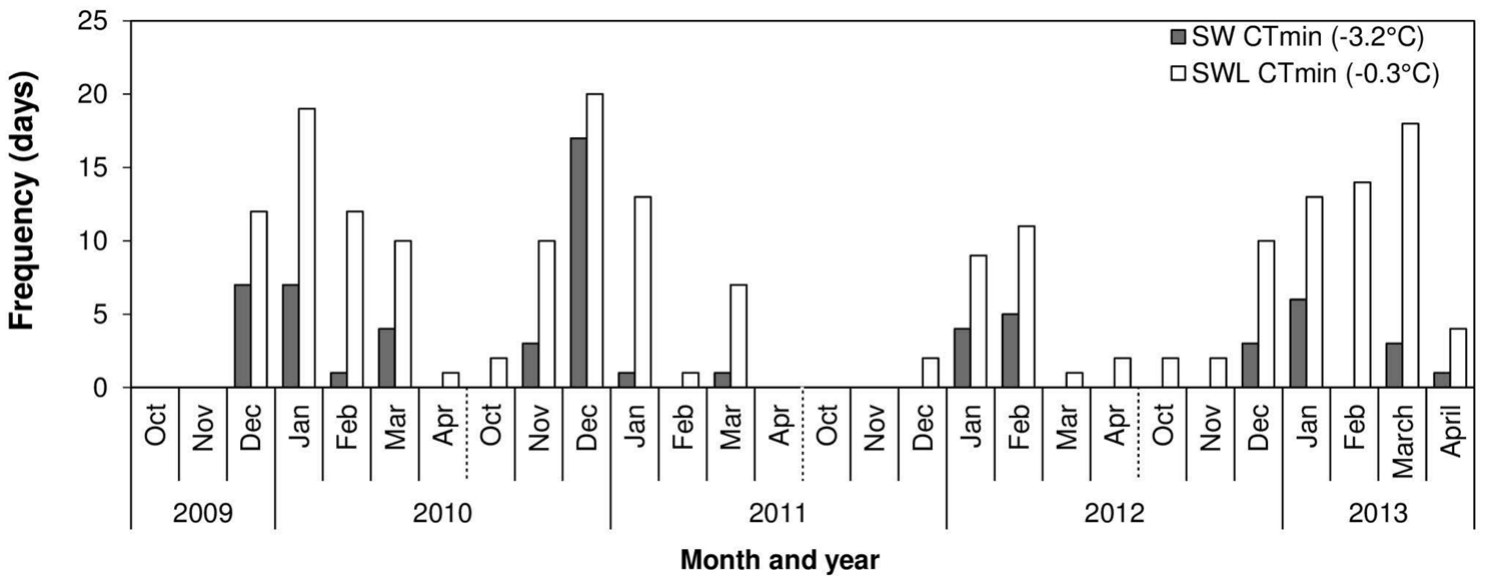
The frequency (total number of days over each month) when temperatures were recorded below the CT_min_ for adult *Calliphora vicina* developed on a diet of sugar, water and liver (SWL; CT_min_ of -0.3°C) or sugar and water (SW; CT_min_ of -3.2°C) only. Air temperature was recorded at an open field site on the University of Birmingham Campus, UK (52.4°N, 1.9°W) from October 2009 to April 2013.

Over the course of the study period (1^st^ October to the 31^st^ April from 2009 to 2013) temperatures were below the CT_min_ for SWL (-0.3°C) and SW (-3.2°C) females for a total of 195 and 63 days, respectively (Fig 5). The frequency of events below the CT_min_ were greatest in December 2010 for both treatments (SWL: 20 d and SW: 17 d).

Temperatures were not recorded below the CT_min_ for either SWL or SW females during the months of October and November 2009 or April, October and November 2010. Temperatures were below the CT_min_ for SWL, but not SW, females in April (1 d) and October (2 d) 2010, February (1 d) and December (2d) 2011 and March (1 d), April (2 d), October (2 d) and November (2 d) 2012. For all remaining months temperatures were recorded below the CT_min_ for both treatment groups.

## Discussion

Late autumn and early winter can be particularly stressful periods of the year for temperate insects. At this time many species are still active and feeding, yet the chances of experiencing unfavourable and rapid diurnal temperature fluctuations are high [3]. This scenario is likely to worsen over the course of this century, as autumn temperatures are predicted to continue increasing [38], and so delaying the onset of diapause[7,39]. Alternatively, warmer winters may result in the early termination of diapause, as a result of depleted energy reserves [40] or ‘false’ termination cues [3]. There is certainly accumulating evidence of earlier spring emergence in many species [41–43], and this scenario brings the risk of exposure to late winter frosts. Thus, understanding how active (and feeding) life stages, not usually exposed to winter conditions, may cope with cold stress is of fundamental importance in predicting changing patterns of insect abundance and distribution under climate change.

The blow fly *Calliphora vicina* represents an excellent model to investigate the impact of winter feeding on cold tolerance because we have clear evidence of climate-driven changes in this species phenology [7,33], and because adult female protein/meat feeding is crucial for egg development/producing the next generation.

Previous work on *C*. *vicina* cold tolerance has largely focussed on larval stages, and noted 100% survival (to eclosion) following 8 d at -4°C in non-diapause larvae, and >70% survival after 7 d at -8°C in diapause larvae [13]. The current study provides important new information on the basal cold tolerance of adult females, and indicates they are considerably less able to tolerate winter conditions. Following just 2h exposure to -6°C, the survival of both SW and SWL females started to decline, falling to approximately 20% for SWL flies after 2h at -7°C, with comparable survival following 2h at -8°C for SW females (Fig 1). These discriminating temperatures (DTemps) are again higher than those of non-diapause and diapause larvae (-10°C and -11°C respectively) [33]. Increased cold tolerance in larvae compared to adult life stages has been observed in several other species, including the kelp fly, *Paractora dreuxi* [44] and the Antarctic midge, *Belgica antarctica* [45]. Evidence from *B*. *antarctica* suggests that selective pressures to develop *in-situ* physiological adaptations may be stronger in relatively immobile larvae, while adults are able to relocate to more favourable micro-climates [46,47]. However, increased cold tolerance in the larval life stage is not a ubiquitous trait, with both *Drosophila serrata* and *D*. *birchii* exhibiting comparable heat and cold tolerance across life stages [48].

The SCPs of *C*. *vicina* females were not significantly altered by feeding (-10.3 and -11°C for SWL and SW flies respectively), indicating that consuming liver does not increase the risk of freezing. This is in contrast with species such as *Bombus terrestris* where feeding can have a dramatic influence on SCPs as a result of introducing ice nucleating agents (INAs) into the gut [11], but may be explained by the fact that *C*. *vicina* consume externally-/pre-digested food that may contain fewer INAs. Certainly within the UK it is unlikely that winter active *C*. *vicina* adults would regularly encounter temperatures below their SCP, however, it is worth noting that a temperature of -13.4°C was recorded on the 20^th^ December 2010. Furthermore, the likelihood of encountering extreme low temperatures in Europe is predicted to increase as winter temperatures become more variable [38,49,50]. In reality, adults are at low risk of succumbing to direct (inoculative) freezing if they have the ability to relocate to warmer microclimates. The impact of feeding upon activity thresholds, therefore, could be crucial in this regard.

Interestingly, both CT_min_ and chill coma temperatures were altered as a result of consuming meat. CT_min_ was reached at -0.3°C and -3.2°C for SWL and SW flies respectively, and these represent thresholds at which adults would no longer be able to move to more thermally buffered microhabitats or escape predation in the field. Over the four year study period (2009–2013) there were 195 days when temperatures fell below the CT_min_ of SWL flies, but only 63 days for SW flies (Fig 5). In December 2010 there was a cumulative duration of 405 hours when temperatures were below the CT_min_ for SWL adults. The mean chill coma temperature of SWL females was -4.9°C (Fig 4), and temperatures below this threshold occurred on 26 separate days between 2009 and 2013. By contrast SW flies had a mean chill coma temperature of -8.5°C, and would have experienced only 9 days below this threshold over the same period. Thus, meat feeding had a clear impact on thermal activity thresholds and, given that chill-induced damage can occur at temperatures above those causing chill coma [23,51], the frequency and duration of chilling injury (and subsequent mortality) would depend on whether females in the field had fed on meat or not. [32,52].

The mechanism by which feeding alters low temperature activity thresholds is not entirely clear, but chill coma is hypothesized to be a consequence of reduced extracellular ion homeostasis at low temperatures [51,53,54]. Increased extracellular K^+^ is certainly a consistent effect of low temperature exposure [55], and can occur very rapidly, e.g. 2 h at -4°C in *L*. *migratoria* [54]. Muscle fibre membrane potential is also dependent on extracellular K^+^ [56], so it has been suggested that increased extracellular K^+^ could cause muscle depolarization during cooling [57]. This is supported by the fact that chill coma recovery coincides with recovery of ‘normal’ K^+^ [54]. Furthermore, impaired chill coma recovery as a result of feeding in the migratory locust, *L*. *migratoria*, was linked to delayed ion (K^+^ and Na^+^) regulation in the muscle and haemolymph [28]. Thus, the high K^+^ content of liver (Table 1) may help explain the elevated thermal activity thresholds of SWL *C*. *vicina*. To test this directly, the diet of SW adult females was supplemented with alanine, which is known to result in cellular K^+^ efflux [29]. However, this did not have a significant effect on CT_min_ or chill coma temperatures, suggesting changes in extracellular K^+^ may not be directly involved. This idea is supported by previous studies in locusts [58], crickets [51] and cockroaches [59] where chill coma occurs prior to significant changes in ionic flux.

Even when inactivated by cold, insects can rapidly enhance their cold tolerance through RCH [35]. The current study indicates that SW female *C*. *vicina* can initiate this response at temperatures as high as 10°C (Fig 2) and under fluctuating thermal regimes (Fig 3) in common with *Eretmoptera murphyi* [60]. The cold tolerance of SW females consistently increased as pre-treatment temperatures were lowered (Fig 2), which again is consistent with other insect studies [60,61]. Interestingly, the cold tolerance response induced under fluctuating temperatures was also sustained during the warmest period of the cycle (20°C), which may prove advantageous for *C*. *vicina* if sudden temperature fluctuations over the winter months become more frequent as predicted under climate change [38]. Temperatures of 0°C are already experienced by active females in late-autumn and early-winter, with adults consistently collected in baited traps between 1^st^ November and 31^st^ December [34], a period experiencing 14, 30, 3 and 14 days with 0°C temperatures in 2009, 2010, 2011 and 2012 respectively. This makes it a real possibility that survival of winter-active *C*. *vicina* is already being enhanced through the process of RCH.

A key result in this study is that meat feeding prevented the RCH response under all treatments as well as the acclimation response under fluctuating conditions (Figs 2 and 3). To our knowledge, this is the first evidence to suggest that access to food can directly influence RCH. It is unlikely that food particles were impeding RCH by acting as INAs, as the SCP of SW and SWL females were not significantly different. As outlined above, it is also unlikely to be the result of changes in extracellular K^+^. An alternative explanation is that an imbalanced diet resulted in metabolic adjustments which influenced cold tolerance. For example, a low protein diet caused increased lipid content in adult female *D*. *melanogaster* [60], and there is known to be a positive correlation between body lipid content and cold tolerance in *Drosophila* sp. [61]. Andersen et al., [62] also identified more rapid chill coma recovery in *D*. *melanogaster* developed on a carbohydrate enriched growth medium, as did Sisodia and Singh [63] with *D*. *ananassae*. Both these studies could help explain the greater cold tolerance of *C*. *vicina* SW flies. However, this theory of increased lipid content under reduced access to protein conflicts with a more recent *D*. *melanogaster* study [64], as well as the idea that compensatory feeding, i.e. over ingestion of carbohydrates in a protein-poor diet [65] results in increased lipid storage and reduced fitness [66,67]. Indeed there is considerable evidence in *D*. *melanogaster* that high levels of dietary sugar negatively affect performance, including chill coma recovery [68]. In addition, all of these previous studies, with the exception of Andersen *et al*., [28], involve extended dietary manipulations that span larval feeding stages, e.g. [62]; or several days during the adult stage, e.g. 6 days [64]. In the current study, however, *C*. *vicina* larval diets were identical, and access to meat was only restricted for 2 days in SW adult females—a very short period of time when considering *C*. *vicina* adult longevity (25 d at 20°C) relative to that of *D*. *melanogaster* (7 d at 20°C). This suggests it is not a responses to nutritional stress per se that caused the differences in cold tolerance.

Another hypothesis is that access to meat in SWL flies diverted resources towards reproduction and away from cold stress responses—thus impacting on RCH, CT_min_ and chill coma phenotypes. Certainly for *C*. *vicina*, and other closely related blow fly species, meat feeding instigates egg development and investment in reproductive processes [4,21,69], and for some female Drosophilidae and Culicidae species even brief access to protein can switch resource allocation towards egg development, diverting resources away from cold adaptation [63,64,70]. Evidence also suggests that meat feeding can induce changes in the composition of the fat body initiating development of reproductive glands in adult male blow flies [69], raising the possibility of feeding impairing cold hardiness mechanisms in adult males, though this requires further investigation.

## Conclusion

Newly emerged adult *C*. *vicina* feeding on a carbohydrate-based diet possess a level of cold tolerance, and the ability to rapidly cold harden, which may allow them to survive the lowest temperatures currently experienced in the UK. These adults cannot persist throughout the many months of winter, however, and so must feed on meat to allow egg development. This study has identified, for the first time, that meat feeding significantly impairs the RCH response in insects, as well as increasing low temperature activity thresholds. Thus, even brief access to protein can shift resource allocation away from stress responses, influencing the frequency and duration of chilling injury. There is increasing interest in patterns of energy trade-offs between stress tolerance and other fitness traits as a result of diet [63,71], but studies in insects to date have mainly focussed on *Drosophila*, which for many reasons is not always the best model to investigate insect stress adaptation [23]. The next step is to characterise the physiological and molecular processes underpinning this change, and ideally in a wider range of species.
